# Reproductive health decision making among nomadic pastoralists in North Eastern Kenya: a qualitative social network analysis

**DOI:** 10.1186/s12978-021-01164-1

**Published:** 2021-05-26

**Authors:** Leah Kenny, Rahma Hassan, Loraine J. Bacchus, Matthew Smith, Bettina Shell-Duncan, Nana Apenem Dagadu, Angela Muriuki, Abdullahi Hussein Aden, Ibrahim Abdirizak Jelle, Beniamino Cislaghi, Mazeda Hossain

**Affiliations:** 1 Faculty of Public Health & Policy, London School of Hygiene & Tropical Medicine, 15-17 Tavistock Place, Saint Pancras, London, WC1H 9SH UK; 2 Present Address: Centre for Women, Peace & Security, London School of Economics and Political Science, Houghton Street, London, WC2A 2AE UK; 3 Institute for Development Studies, University of Nairobi, 4 Harry Thuku Rd, Nairobi, Kenya; 4 The Business School, Edinburgh Napier University, Edinburgh, EH14 1DJ UK; 5 Department of Anthropology, University of Washington, 314 Denny Hall, Box 353100, Seattle, WA 98195-3100 USA; 6 Save the Children US, 899 North Capitol St NE, Suite 900, Washington, DC 20002 USA; 7 Save the Children Kenya, Matundu Close, Off School Lane, Westlands, P.O. Box 39664-00623, Nairobi, Kenya

**Keywords:** Social network, Nomadic pastoralist, Family planning, Reproductive health, Social norms

## Abstract

**Background:**

To our knowledge, no studies exist on the influence of nomadic pastoralist women’s networks on their reproductive and sexual health (RSH), including uptake of modern family planning (FP).

**Methods:**

Using name generator questions, we carried out qualitative egocentric social network analysis (SNA) to explore the networks of four women. Networks were analyzed in R, visuals created in Visone and a framework approach used for the qualitative data.

**Results:**

Women named 10–12 individuals. Husbands were key in RSH decisions and never supported modern FP use. Women were unsure who supported their use of modern FP and we found evidence for a norm against it within their networks.

**Conclusions:**

Egocentric SNA proves valuable to exploring RSH reference groups, particularly where there exists little prior research. Pastoralist women’s networks likely change as a result of migration and conflict; however, husbands make RSH decisions and mothers and female neighbors provide key support in broader RSH issues. Interventions to increase awareness of modern FP should engage with women’s wider networks.

## Plain language summary

Few studies have asked nomadic women in Kenya to name the important individuals in their lives when it comes to making reproductive and sexual health decisions, including their use of family planning. These important individuals are described as a woman’s “network”. We used a survey and open-ended interview format to identify the individuals in four nomadic women’s networks (“social network analysis”). Data was analysed in R and we created a visual map of these networks. Women named 10–12 individuals. Women’s husbands made reproductive health decisions and did not approve of modern family planning use. Apart from their husbands, women did not know who in their network approved of their use of family planning. Female neighbors and mothers provided important support to women. Interventions to increase awareness of modern FP should engage with everyone in a woman’s network.

## Background

Nomadic and semi-nomadic pastoralists make up the majority of the population living in Wajir and Mandera counties on the border with Somalia, and represent some of the most marginalized populations in Kenya [1–3]. These sparsely populated arid lands are characterized by low and erratic rainfall and high temperatures, making them challenging for agriculture [4]. Instead, the livelihoods of these groups are centred on livestock production (in particular camel, cows, and goats) as a source of food and income. These ethnically Somali pastoral groups practice seasonal migration, influenced largely by the needs of their livestock, and often in response to persisting intercommunal conflicts, terrorist attacks, and periods of protracted food insecurity [5]. As a result, there exists very limited accurate and up-to-date census data for these populations, who are often omitted from national surveys [6, 7]. Poor health and educational indicators for North Eastern Kenya include that 93% of women have no formal education and over two-thirds of women do not receive any antenatal care, figures that are likely worse amongst nomadic pastoralists [8, 9]. Nomadic pastoralist women and men are particularly vulnerable to infection and disease, with high maternal and child mortality resulting from often preventable causes [10].

There are an estimated 50 million nomadic pastoralists in Africa [11], and in the horn of Africa this is estimated to be 20 million [12]. While they represent a heterogeneous group, they share common livelihood (livestock rearing) and related mobility patterns, at times crossing national borders, and have limited access to health care [10, 13]. The small number of studies on the health of nomadic groups attribute high mortality and morbidity amongst nomadic groups, when compared with settled populations, to poor nutrition, poor access to formal education, and the inaccessibility of existing health care, including reproductive and sexual health (RSH) services, due to a range of cultural, political, economic, and structural factors [4, 14–17]. Half (61%) of married women in Kenya currently use modern methods of family planning (FP), compared with just 2% of women in Wajir and Mandera counties [9, 18]. Low modern FP use in Kenya and elsewhere has been attributed to a range of factors that influence demand for and access to quality RSH services, including average distance to health facilities, fear of side-effects, gender norms, partner opposition or opposition from other community members, and religious and cultural barriers to modern FP [17, 19–24].

Social norms can help explain reproductive health decisions, including the use of modern FP to space children [25–27]. According to social norms theory, individuals follow unwritten and unspoken rules that are shared amongst people in their reference group [25, 28, 29]. These rules are mutual behavioral expectations and are sustained, amongst other things, by anticipation of approval or disapproval for, respectively, complying or not with them [30]. They can be defined as descriptive (beliefs about what others do) or injunctive (beliefs about whether others will approve) [31]. Gender norms are social norms that specifically define what is expected and acceptable for a woman or man in a given society [32]. Unequal gender norms in particular, tend to disadvantage women, who are often not in charge of their own RSH decisions, including their use of modern FP methods [33]. Amongst pastoralist communities in Kenya, women make decisions related to pregnancy and childbirth with others, influenced by the perceived normative approval or disapproval among family members, peers, and other community members that make up their reference group [25, 34–37]. RSH decisions are at times made by or with husbands, mothers-in-law, or mothers-in-law [35, 38], depending on for example, a woman’s autonomy at the household level [39]. During periods in which husbands are away herding, other individuals may play a role in shaping nomadic pastoralist women’s decisions, including a woman’s parents-in-law, brothers-in-law and her own parents [40].

We carried out a qualitative egocentric social network analysis (SNA) through interviews with two nomadic and two semi-nomadic pastoralist women living in Wajir and Mandera. Network studies have proven useful in identifying the spread of health information and behaviors amongst populations [41, 42], including those relating to RSH [43–45]. While our focus was women’s use of modern FP, as defined by the Demographic Health Surveys (DHS), due to the sensitive nature of discussing FP in this setting we included a broad definition of RSH encompassing: timing of first born; birth spacing; and access to health services. We set out to examine who is important from a pastoralist woman’s perspective, identifying key influencers and decision makers on issues pertaining to RSH. This paper has four aims, to: (1) explore pastoralist women’s views on modern FP; (2) describe the social support networks in which pastoralist women are embedded; (3) identify key people within these networks who influence RSH decision making; and (4) explore perceived versus tangible support of modern FP.

## Methods

### Study sites and participants

Participants in this study were nomadic women and men from Mandera (N = 11) and semi-nomadic pastoralist women and men from Wajir county (N = 12). There is high variability in mobility and sedentarism amongst pastoralist populations, particularly as they adapt to ecological and economic demands [46, 47]. We distinguished between these two nomadic community types based on their levels of sedentarism (semi-nomadic populations settled for longer periods of time) and their livestock (nomadic communities predominantly herd camel, while semi-nomadic communities herd cattle), as defined by Save the Children Kenya field staff. The composition of nomadic and semi-nomadic settlements in these areas are dynamic and not captured in national census data [48]. A semi-nomadic or nomadic *bulla* (village) may, for example, be dispersed over a 30 km radius. It was estimated that study sites for this research had populations of between 350 and 400 individuals, with around 100 women of reproductive age. Community leaders, because of their influence in the community, were key to recruiting participants. In each of the study sites, community leaders helped to purposively identify women of reproductive age (19–49 years) who were willing to be interviewed. Four women were chosen by the community leaders to be seed informants, two each from a nomadic and a semi-nomadic community. In the initial interviews, these women served as “ego” or the interviewee providing names of and information about people within their social circles; these social contacts who were named are referred to as “alters.” Each ego listed individuals of importance to them, with no limit on the number they could name. The five most influential alters were identified, and they, in turn, became secondary respondents (egos) in the following interviews. A total of 23 individuals were interviewed, as we were unable to contact one alter.

### Study design

To explore the social networks in which nomadic and semi-nomadic women were embedded, and how these shaped social norms related to RSH, we carried out qualitative egocentric SNA. The ego-centric approach to data collection is useful in this setting as there is no complete list of relevant individuals, therefore this approach aids in defining the network boundary [49, 50]. Qualitative egocentric network interviewing methods, first developed in the 1950’s in the field of anthropology, have recently resurged in popularity, either as stand-alone methods or in combination with quantitative SNA methods, to *illuminate the arenas of social interaction that shape or enforce social norms, as well as the composition of network partners, patterns of influence, and the enforcement of social norms that uphold or challenge prevailing practices* [51, 52]. In this study, we adapt the qualitative methods employed by Moreau and Shell-Duncan [53] to study social network influences on female genital cutting in Senegal. Adapting this approach for the study of FP in Kenya, we developed a semi-structured interview protocol that involved several steps. First, interviews with primary respondents centered on in-depth descriptive accounts of their own marriages and childbirths. Second, women were asked a series of generalized name generator questions, adapted from those used in other settings, to build a list of important people, with no limit on the number they could nominate [54]. Respondents were all asked the same interview questions. Interviewers made notes on the gender and relationships of all individuals named. Third, informants were then invited to identify key ‘influencers’ and ‘decision makers’ in relation to their RSH and modern FP use. This involved asking women to think who from their name generator list contributed to making decisions around their RSH, and ego could add individuals not already listed. These were labelled decision makers. Women then indicated the top five most influential people in relation to their RSH. Many empirical studies apply some form of limits on the number of alters named in response to name generators [55]. Existing literature suggests five names is sufficient and cost-effective in name generator approaches to collecting network data [56–58]. Listing four to five alters is sufficient to observe the true connections of the ego, while more names likely leads to redundancy [59] and egos are unlikely to list more than five [60–62]. Table 1 provides examples of questions asked during stages two and three. The five influencers were ranked from most to least influential, and ego explained why she had chosen each individual. Finally, women were asked if they thought each influencer was supportive of their modern FP use, and each response was recorded.

**Table 1:** Name generator, decision maker, and influencer questions

Question type	Example question
Name Generator	Who is most important to you? Who do you talk to if you are worried or upset? Who do you leave in charge of your livestock? Who do you leave in charge of your children?
Decision maker questions	Who was involved in decisions around the timing of your first child? After your first child, who participated in decisions around how long to wait to have another one? Who participates in decisions around how many children to have? Who participates in decisions around how much or how long to breastfeed for?
Influencer questions	Who are the people who have the most influence over the number of children you had (or will have)? Can you rank these individuals from least to most influential?

The next round of interviews was conducted with each of the five influencers reported by the primary respondents. These secondary respondents were asked the same name generator questions, thus creating their own immediate egocentric networks and the wider network. This is referred to as a 2-step network [63], which includes those directly connected to ego and individuals indirectly connected through their alters (or 1-step partners). Influencers were also asked if they had contributed to ego’s decisions regarding RSH. Finally, they were asked if they were supportive of ego’s use of modern FP. This was compared with what ego said in her interview, but they were not informed of each other’s responses to questions.

The egocentric approach therefore had two stages: (1) semi-structured interviews with individual women (the egos); and (2) shorter semi-structured interviews with the top five influencers identified in stage one. We chose not to extend beyond the secondary respondents’ networks as we were solely interested in the local network of our primary respondents [64].

### Data collection

In November 2018 semi-structured interviews were carried out in same-sex pairs. These were conducted in either Somali or Borana. One interviewer conducted the interview, whilst the other had a notetaking role, filling out paper-based name generator forms. Upon completion of the interview, name generator forms were quality checked together with ego to ensure the correct influencers, decision makers and their respective relationships had been recorded. If women clarified a relationship (e.g., that a female neighbor was also a sister-in-law) this was added to the form. It was important to check that individuals listed did not use any other names, so they could be correctly identified. Neighbors are defined as individuals residing next to each other in semi-permanent or temporary *bullas* (villages). Finally, interviewers did their best to clarify relationships that were described, distinguishing between ‘true’ and ‘fictive’ kin, as individuals were often given familial ties irrespective of their relationship [65, 66].

Community leaders helped identify the five influencers who were then invited to interview. These interviews were also carried out in same-sex pairs, in participants’ preferred language (Somali or Borana), and near homesteads, to ensure participants were comfortable. Where influencers could not be located for any reason, interviewers attempted to contact them by phone.

### Facilitator training and ethical considerations

Interviewers were originally from Wajir or Mandera counties (but not from the study sites) and were fluent in Borana, Somali, and English. Interviewers received training in social norms and SNA methods over 5 days, prior to data collection. The training provided an opportunity to explore terminology and the ways ‘decision maker’ and ‘influencer’ could be interpreted in the different languages. It was agreed that ‘influencers’ were important people whose opinion the women valued when making decisions, but who did not necessarily make the decision with/for them. This definition was trialed within the research team, agreed upon, and included in the interview guides. Daily debriefs conducted with the whole research team were used to discuss challenges as they arose. All interviews were audiotaped and later translated and transcribed. Initial transcripts were quality checked by the data collection coordinator, who was fluent in both Somali and Borana. Oral consent was obtained prior to conducting all of the semi-structured interviews, and permission to speak with identified influencers was obtained from the ego upon completion of her interview. Results for this paper form part of a larger qualitative study on family planning amongst nomadic and semi-nomadic communities in North Eastern Kenya. Participating communities received tea and sugar (local commodities deemed appropriate by field staff), distributed by community leaders.

Ethical approval for this study was obtained from the London School of Hygiene & Tropical Medicine (Ref: 16109) and from Amref (Ref: P542/2018).

### Data analysis

Data from the hand-written name generator forms were entered into Excel and quality checked against interview transcripts, particularly where handwriting was illegible. Data analysis occurred in two concurrent stages: we conducted a framework qualitative data analysis utilizing the interview transcripts [67], and egocentric SNA using the name generator forms to create network maps. The combined networks of primary and secondary respondents were used to create sociograms (visual displays of the networks), and compared across study sites.

1. Modern family planning definition
  We used the Demographic Health Surveys (DHS) definition of modern family planning which includes: female and male sterilization; contraceptive pill; Injectable; implant; diaphragm; lactational amenorrhea method (LAM); standard days method (SDM); male and female condom; intrauterine contraceptive device; and emergency contraception [68].
2. Qualitative analysis
  A framework approach was used to analyze the semi-structured interview data. Initially, we familiarized ourselves with the interviews, making note of any interesting ideas or concepts that emerged. We then created a matrix, comparing the four individual egos answers to specific and relevant sections in the interview. We did the same for the five influencer interviews. The matrix included answers to questions in relation to child spacing (including obstacles to using modern FP) and to reproductive health decision makers (including reasons why certain individuals were important). We then looked for themes across both ego and influencer interviews.
3. Egocentric SNA
  Egocentric network maps were created using R and Visone (version 2.17) [69]. Each map included both ego’s immediate network (those individuals she had named as important), and the wider 2-step network (individuals named by each of ego’s influencers). For each network map, we initially created a node list (all the individuals in a network) and an edge list (relationships between individuals) before adding attributes to these nodes (e.g. socio-demographic information) and edges. The final data frame was converted into a graph object, and we used the iGraph package in R to calculate basic network statistic [70]. Data frames created in R were opened in Visone, to visualize the final maps.

## Results

### Study sample

Our sample included four egos (two semi-nomadic and two nomadic women). EGO1 and 2 were from a nomadic community, while EGO3 and 4 were from a semi-nomadic community. We interviewed each of the women’s top five influencers, and the total study sample included 23 men and women (we were unable to interview EGO1’s husband, who was away herding at the time of data collection). Table 2 presents the attributes of our study sample, including the egos (n = 4) and each of their top five influencers (n = 20). Three egos were aged between 19–35 and one between 36–49 years old. All four women had married before the age of 18, and they had given birth to their first child between the ages of 15 and 22, giving birth to 8–10 children. Finally, as anticipated, the women were all Muslim and spoke Somali.

**Table 2:** Attributes of four women (egos) and their top five influencers

EgoID	Ego attributes						Top five ranked influencers (*ranked from most to least important*^a^)					
	Ethnicity	Language	Age *(category)*	Number children	Age married	Age at first child	Relation	Sex	Age	Number children	Age married	Age at first child
EGO1 (nomad)	Dogodia	Somali	40 (*36–49*)	10	15	17	Husband	M	–	–	–	–
							Neighbour	F	36–49	10	14	17
							Neighbour	F	19–35	7	13	13
							Neighbour	F	19–35	3	15	15
							Neighbour	F	19–35	2	19	–
EGO2 (nomad)	Dogodia	Somali	30 (*19–35*)	8	16	22	Brother	M	–	16	23	24
							Husband	M	36–49	11	23	25
							Cousin	F	19–35	5	15	20
							Religious Leader	M	36–49	3	20	–
							Cousin	M	19–35	0	19	NA
EGO3 (semi nomad)	Ajuran	Somali and Borana	35 (*19–35*)	9	14	–	Cousin	F	19–35	5	19	22
							Brother-in-law	M	19–35	7	25	26
							Cousin	F	19–35	7	16	20
							Husband	M	50–69	13	19	22
							Cousin	F	36–49	8	13	–
EGO4 (semi nomad)	Ajuran	Somali and Borana	30 (*19–35*)	8	15	15	Sister-in-law	F	19–35	7	15	17
							Aunt	F	70+	11	19	20
							Neighbour	F	36–49	5	16	20
							Aunt	F	36–49	10	14	16
							Neighbour	M	19–35	4	19	21

^a^Influencers have been listed in their order of importance, according to the women (1 = most important, 5 = least important)

Amongst the influencers, women were married at similar ages (13–19 years), had their first child before they were 22 and had given birth to 2–11 children. Men in our study were married later, the youngest married at 19 years, the oldest at 25. As a result, they had their children later yet had up to 16 children.^1^

Table 2 also shows the relationship between ego and each of her influencers, as described by ego. There was variation in the types of relations women had with those they named as influential in their networks, these included: neighbors (female and male); cousins (female and male); husbands; aunts; a brother; a brother-in-law; a sister-in-law; and a religious leader. We measured how similar the women were to their influencers. All of the women had more female influencers than male, except for EGO2(nomad). Women had a mix of younger and older influencers, and only one of the women (EGO3(semi nomad)) had the majority of her influencers in the same age category as her.

### Women’s views on modern family planning

When asked about personal modern FP use, all four egos had heard of some modern methods. In particular, they named the injection and contraceptive pill. Women said they had not used “modern methods” obtained from health centers, but that they did practice breastfeeding. For instance, EGO4(semi nomad) said, *I didn’t use any child spacing methods […] I stop breastfeeding when I get pregnant* and EGO3(semi nomad) said *If you breastfeed for 3 years, that itself is a child spacing method*. Others spoke of other child spacing methods such as abstinence (EGO2(nomad)): *I did not use any [modern methods], but when our husbands are here, we ask them to stay away for some time*. However, EGO3(semi nomad) (aged 35), reflected on how she was not aware of modern FP methods when she was younger, *During those days there was nothing of that sort; it’s just recently that we have heard of injections and pills to space children*, but like other women in this study she was not directly asked about her personal use and did not offer this information.

The four women described how women in their communities either did not use modern FP, or kept their modern FP use hidden. For example, EGO2(nomad) said, *No, we do not use [modern family planning] here* and similarly EGO1(nomad) said, *I have not heard of women going to get this child spacing methods, and I have not seen women going to the hospital to get them.* In contrast, EGO3(semi nomad) explained how if women used modern FP, they hid their use to avoid being identified and the possible repercussions of this. She said that in her semi-nomadic community *Women keep [modern FP] to themselves, they hide so that no one knows; they even hide from their husbands.*

Modern FP was described as going against religious beliefs and God, who was ultimately in charge of spacing children as seen in EGO2(nomad)’s comment*, Only God can space children.* However, non-use or negative attitudes towards modern FP were also due to its association with infertility, as EGO4(semi nomad) said, *I have heard of those pills you take if you never want to have a child* and similarly EGO2(nomad), *Some people say those methods make us infertile.*

### Women’s wider social networks

Table 3 presents a summary of each of the women’s networks. It includes each of the four ego’s immediate networks, in addition to the wider 2-step network (including alters’ networks). The complete network ranged in size from 37 to 70 individuals. The social ties indicate the number of unique relations in the network, and as such were larger than the network size as they included where multiple influencers had named the same individual, but not when there had been reciprocity in naming (e.g. A named B, and B named A). The number of social ties ranged from 42 to 77. The larger networks had more social ties and were explained by a greater number of individuals named by the five influencers (which ranged from three to 30 people). Male influencers named more individuals, compared with females, for example, a male influencer in EGO2(nomad)’s network named 30 individuals. Density captures the level of connectivity in the network, the ratio of observed ties to all possible ties [71]. The network connectivity is consistent across all four networks. Triads represent when three actors in the network are connected (A links to B, B links to C and C links to A, i.e. the friend of my friend is my friend) [72]. In these networks we observed very few triads in these influencer networks.

**Table 3:** Summary of structure and composition of wider networks

	EGO1 (nomadic)	EGO2 (nomadic)	EGO3 (semi-nomadic)	EGO4 (semi-nomadic)
Structure of wider network^a^				
Size (not including ego)	37	70	43	42
Social ties	42	77	46	53
Density	0.068	0.031	0.054	0.058
Ego degree^b^	14	15	13	11
Out degree	*10*	*12*	*12*	*10*
In degree	*4*	*3*	*1*	*1*
Number of triads	5	5	6	4
Composition of wider network				
Females (%)	19 *(51%)*	19 *(27%)*	16 *(37%)*	21 *(50%)*
Males (%)	18 *(49%)*	51 *(73%)*	27 *(67%)*	21 *(50%)*
Relationship type^c^				
Family	23 (55%)	46 (60%)	31 (67%)	40 (75%)
Mother	3	4	1	4
Father	1	4	1	0
Husband	5	2	3	5
Wife	0	5	1	1
Brother	2	12	5	4
Sister	4	4	1	4
Son	1	0	0	2
Daughter	2	1	0	4
Female cousin	0	1	8	6
Male cousin	1	6	3	4
Aunt	0	1	2	4
Uncle	–	3	0	3
Other	4	3	6	5
In-laws	None	6 (8%)	5 (11%)	6 (11%)
Mother	–	–	1	1
Father	–	–	1	–
Brother	–	4	2	1
Sister	–	2	1	1
Son	–	–	–	–
Daughter	–	–	–	2
Other	–	–	–	1
Community member	12 (29%)	12 (16%)	7 (15%)	7 (13%)
Female neighbor	12	1	2	3
Male neighbor	–	2	–	2
Female friend	–	1	1	–
Male friend	–	5	3	2
Other	–	3	1	–
Community official	7 (16%)	13 (17%)	3 (7%)	None
Village chief, elder, chair	6	7	3	–
Religious leader	–	6	–	–

^a^Number of unique individuals in the network (2.0 degree), including individuals named by ego and her alters

^b^Degree refers to the number of ties an actor has in the network, degree can be separated into: in degree (number nominations an actor receives) and outdegree (number of nominations an actor makes)

^c^Frequency that certain types of relationships came up in the wider network. Relationships were defined by the women and men interviewed (ego or her influencers)

Looking at the wider networks, EGO2(nomad) and EGO3(semi nomad) had more males (73% and 67% respectively) than females, while EGO1(nomad) and EGO4(semi nomad) had similar numbers of males and females. The degree, which refers to the number of ties an actor has in the network, was higher for the two nomadic women, as they were mentioned more often by their influencers. Family members were the most prevalent relationship type. Within this, husbands, mothers, brothers and sisters were mentioned across the four networks. Among other “community members” female neighbors were mentioned frequently, while “community officials” were more present in the nomadic compared with semi-nomadic networks.

### Women’s immediate networks

Women named 10–12 individuals in their immediate network. The semi-nomadic women had a higher proportion of females, while the nomadic women had either similar numbers, or fewer females. Similar to the wider network, three of the women named mostly family members in their networks, while EGO1(nomad) had community members (e.g. female neighbors) and community officials (e.g. village elders). Finally, all networks included husbands and mothers, while only one network (EGO4(semi nomad)) included her mother-in-law.

**Fig. 1:**
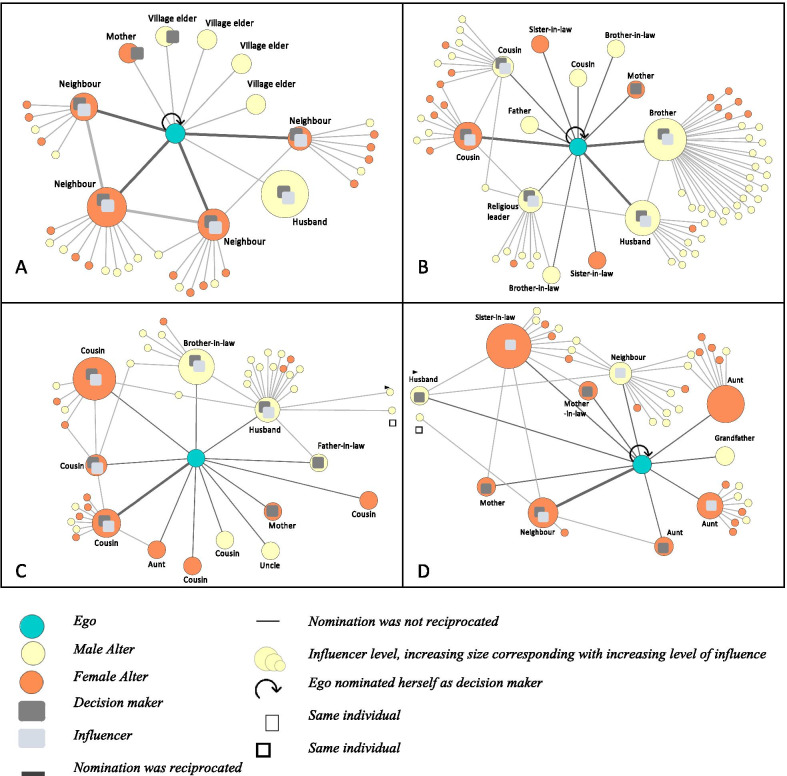
Women’s social network maps. Egocentric maps for each woman (ego) presented individually, with ego at the centre of their social network. The maps include the relationships between ego and those in her network. Individuals named as decision makers and the five influencers in issues relating to RSH have been highlighted. Maps also include the size and gender make-up of the wider network. **A** EGO1 nomadic; **B** EGO2 nomadic; **C** EGO3 semi nomadic; **D** EGO4 semi nomadic

Figure 1 captures women’s social networks in greater detail. The maps also provide an overview of the women’s wider-2-step network. An in-depth analysis of alter-alter ties is beyond the scope of this paper, and we therefore focus on the immediate networks We present each network separately, however, as Fig. 1A, B show, two male individuals act as a bridge between the semi-nomadic networks. These maps highlight the key decision makers and top five influencers concerning reproductive health, and importantly their relation to ego and position within each of the networks. For three of the four women, all of the influencers were also decision makers (in addition to two individuals that were only decision makers). EGO4(semi nomad) clearly distinguished between influencers and decision makers. While there was variation between the women’s social networks, there was overlap in the role of the mother and husband across the networks. These relationships are explored in greater detail below.

#### The role of the mother

Mothers were decision makers. Women described how their mothers were important decision makers before they left their natal home to get married, or while they were still living close to their mothers. Their mothers helped make decisions before and during their first pregnancy, for example, EGO3(semi nomad) described how she made these decisions with her mother while they were still living together: *I lived with [my mother] then in the same compound, and I choose her because we lived together and I trusted my children with her.* Interestingly, her mother was also a traditional birth attendant and had assisted in EGO1(nomad)’s first birth. EGO1(nomad) described the shift from making decisions with her parents, to making them with her husband: *[M]y parents, I talk to them first before deciding anything. The rest come after them. Mothers and fathers take care of the children and before you consult with anyone else, you decide with them first. […] After marriage, it is my husband whom I decide with*. That the father was no longer alive at the time of the interview could explain why only her mother was named in the network.

#### The role of the husband

Husbands were decision makers in all three of the ego networks in which they appeared, and were also influencers in three (see Fig. 1A–C). EGO1(nomad) and EGO2(nomad) named their husbands as one of the most important people to them, and the most or second most influential in their network. EGO2(nomad) described how her husband was the sole decision maker in issues relating to child spacing: *[H]e is the only one who I agree with. It is just him, because nobody else has the right over my body.* This was also true for EGO3(semi nomad), who despite naming her husband late in the interview, said he made decisions relating to child spacing and the number of children to have: *It’s all my husband and no one else*. In the network where the husband was a decision maker, but not an influencer (Fig. 1D), EGO4(semi nomad) described how decisions were shared between her, her husband, and her mother-in-law as she stated, *I had left my mother after marriage; [my mother-in-law] was like a mother to me,* revealing how her mother-in-law assumed a similar maternal parenting role for child spacing discussions once EGO4(semi nomad) left her natal home.

#### Pastoralist women as decision makers

Three of the women named themselves as decision makers in issues relating to breastfeeding, as depicted by the loop in the network maps. EGO2(nomad) described herself as the sole decision maker in issues relating to breastfeeding and spacing, *[I]t was all me who decided to breastfeed, no one made that decision with me*. This was also true for EGO1(nomad), and EGO4(semi nomad) said she also played a role in deciding how many children to have. In instances where the women were also named by their top five influencers (illustrated by the thicker lines in the network images), this was because they provided material and emotional support to these individuals, for example looking after their children and livestock.

### Perceived and tangible support of modern family planning

**Fig. 2:**
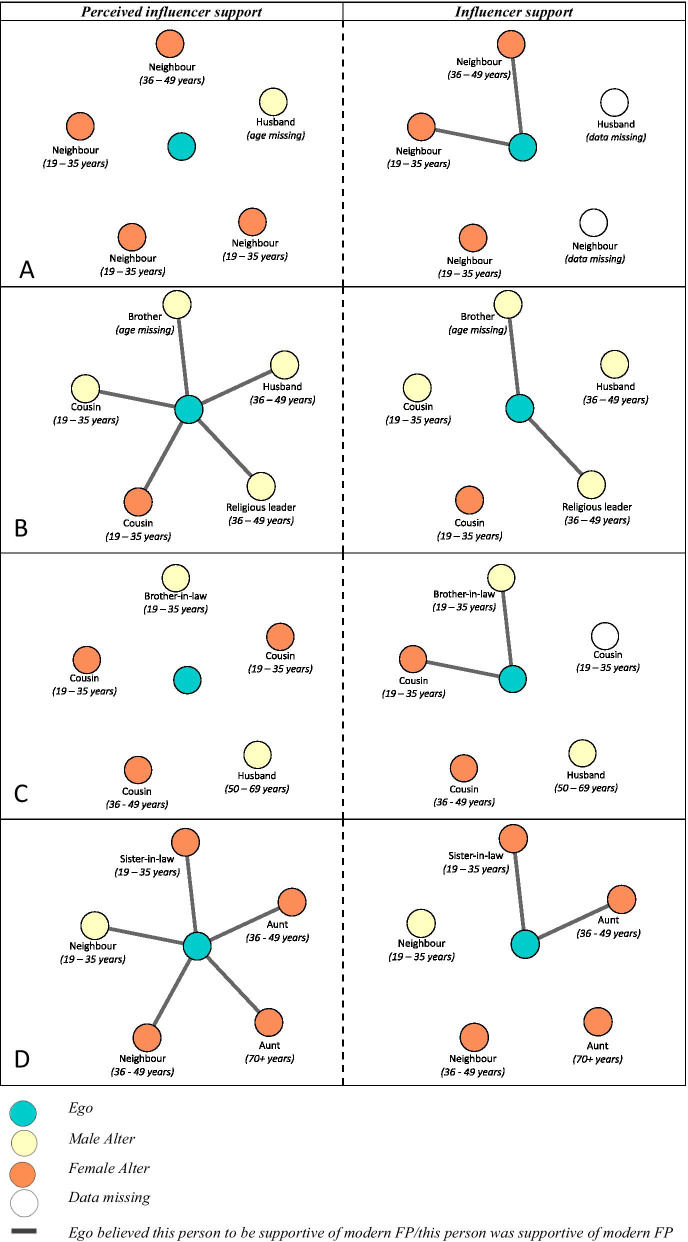
Comparing perceived and tangible support for modern FP amongst five influencers. Who women (ego) believed would support their use of modern FP (left hand side) compared with their response to whether they supported ego’s use of modern FP. A line between ego and an influencer indicates either perceived or tangible support for modern FP. No line indicates no support (unless data is missing)

Figure 2 captures the four egos and their five influencers. It depicts egos’ description of influencers support of their modern FP use (“perceived support”), and what the influencers said in their interviews (“tangible support”). The images show that perceived and tangible support often conflict, highlighting that none of the four women knew whom within their social networks would support their use of modern FP. Few patterns emerged by age or nomadic type; however, all three husbands were unsupportive of its use.

EGO1(nomad) and EGO3(semi nomad) (Fig. 2A, C) believed that none of their influencers would support their use of modern FP, although, two influencers were supportive. For example, two of the younger female neighbors said they would support EGO1(nomad)’s use of modern FP, expressed positive attitudes towards child spacing, and believed that EGO1(nomad) should make her own FP decisions. For example, one said: *I would support her because it is good for us. It is good because child spacing helps us, it is good for us and our children*. The other showed she would be willing to actively support ego if she chose to use modern FP: *[If] that is what she wanted, I would encourage and ask her to go because she has already decided herself anyway. If I too wanted to use [modern family planning] we would go together*. Similarly, EGO3(semi nomad)’s female cousin (19–35 years) and brother-in-law (19–35 years) said they would actively support her if she wanted to use modern family planning. For example, the brother-in-law said, *[If] she wants to use modern child spacing methods, I will escort her so she gets those jabs [contraceptive injection], I will tell my brother, and I will strongly support*. This showed he would both help source modern FP and encourage his brother (ego’s husband) to support.

EGO2(nomad) and EGO4(semi nomad) (Fig. 2B, D) said that all five influencers would support their modern FP use. Two individuals in each of their networks said they would support the use of modern FP. The religious leader was supportive of EGO2(nomad) if modern FP was used solely for child spacing purposes, *If she wants to space her children, then I will support her if her children are not well spaced*, while her brother (age missing) said he supported modern FP use to improve child health, *Yes, I would support her because if women get pregnant immediately after 40 days, then the older children get less attention and become sickly*. The brother’s network map and interview revealed he shared a social tie with a male nurse working in the nearby town. EGO4(semi nomad)’s sister-in-law and aunt were supportive, but described they were not aware of the different modern FP methods. For example, the sister-in-law (19–35 years) said, *I would support her if she feels she has had enough, and we will definitely talk about [modern FP], if it’s possible we will do it, but if not I will advise her to stay away from it*, as such FP could be used under certain circumstances.

Influencers who were unsupportive described modern FP was against their religion and God. For example, EGO1(nomad)’s female neighbor said: *[I] would advise her not to compete with God* and EGO3(semi nomad)’s husband said: *I won’t support her because if God gave you children, why would you stop?* Others felt that the decision needed to be made solely between a husband and wife, for example EGO2(nomad)’s male cousin said: *I cannot discuss such things because I am not married to her*. Finally, the eldest of the influencers (EGO4(semi nomad)’s aunt) said that women can only bear children for a short amount of time, *[T]he duration women bear children is limited*, thereby implying that women should continue to give birth and not use modern FP methods to limit or space children.

## Discussion

A woman’s ability to space her children through her preferred method of FP, is affected by multiple factors including demographic characteristics, the availability of and access to RSH services, and the social and gender norms within her community. Norms related to child spacing and FP are often upheld by important individuals in a woman’s reference group, which can in turn influence her use. Women in this study distinguished between breastfeeding and other medical methods obtained from health services (predominantly the injection and contraceptive pill). As such we used the term modern FP throughout this paper to refer to these medical methods, while recognizing the various methods as defined by the DHS. We found evidence for a norm against modern FP and this was both descriptive (that no one in the community uses modern medical FP), and injunctive (women hide their use of modern FP, possibly to avoid negative social sanctions). Positive attitudes towards breastfeeding point to an opportunity for information and counselling around exclusive breastfeeding/lactational amenorrhea method (LAM). While we were unable to ask women about personal use due to the sensitive nature of the topic in this context, their awareness of modern FP methods is likely a reflection of the Kenyan government’s commitment to address inequitable access to FP at the county level. While we anticipated women and men to be isolated (particularly nomadic communities), this shows that local health services and messaging have reached these communities, and there is likely interaction between communities. Nevertheless, norms, religion and fears around modern FP likely prevented pastoralist women from reporting their use.

This study is notable for its use of qualitative interviews, which allowed rich descriptions of pastoralist women’s immediate networks. While studies often assume a reference group, we allowed ego to identify her own network [73]. Similar to other network studies, pastoralist women in our study named 10–12 individuals [74]. Except for one woman who named more males than females, networks had either equal gender distribution, or more females. All four networks included husbands and mothers, and the majority of individuals were family members (except one network, where female neighbors were more prevalent). It was difficult to make comparisons since each egocentric is unique and the sample size is small. Nonetheless, we found female relatives and neighbors formed a large part of the network and provided support in areas related to reproductive health and FP (for example, advising on breastfeeding periods). Their key role in the networks could be due to women’s proximity to each other, and the fact that women rely on nearby support during pregnancy, and to care for their children. While men and husbands were involved in RSH decisions, such as whether to have another child, they may be less involved in day-to-day decisions (such as visiting a health facility). Finally, while other studies show community leaders are key influencers in FP issues [75–77], religious and community leaders formed part of the reference group for nomadic pastoralist women, but not semi-nomadic pastoralist women.

The women in this study all described their husbands as RSH decision makers, which was also confirmed by husbands and influencers in the networks. Studies show there is low uptake of modern FP when men dominate decision making [78, 79], while male partner support has been associated with increased use [80, 81]. Three women in this study said they made child spacing decisions on their own, reflecting some degree of autonomy over their RSH, which has been associated with positive health outcomes [82]. While a mother-in-law only appeared in one network, all four women described their mothers as decision makers, and as important individuals in their lives, despite no longer living near to them. It is likely that mothers were decision makers in issues relating to pregnancy and childbirth before the women left home, but not necessarily in relation to modern FP.

Pastoralist women did not know who in their network was supportive of modern FP. Two women reported that all of their influencers supported modern FP use, while the others reported that none did. Often however, these perceptions were incorrect, and influencers reported differently to their egos. We cannot comment on how this affected women’s personal use of modern FP, however, we anticipate that women who believe their reference group to be supportive, are more likely to access and use modern FP if they desire to [79, 83, 84]. Opening up discussions around FP methods would allow women to know who in their reference group was supportive of modern FP use. As women named a diverse range of influencers in terms of their relationships to ego, we found few patterns across influencer type. However, husbands were never supportive of its use. We anticipated that males would in general be unsupportive, however, male relatives (a brother and a brother-in-law) and a male religious leader were supportive. Similar to other studies, religious leaders supported modern FP if it was for spacing, but not to limit the number of children a woman had [36]. We found that the brother shared a social tie with a male nurse working in a nearby town, which could explain his acceptance, despite the norm and negative attitudes towards modern FP. This highlights the importance, not only of who is in your immediate network, but individuals in the wider network. Meanwhile, support for modern FP amongst female relatives of the same age was more nuanced, as seen in the example of the sister-in-law who was unsure of her support yet believed having too many children was a burden for women.

Data collection for qualitative egocentric SNA is time consuming, taking up to one working day to for each network. In addition, it requires accurate notetaking and record keeping. Our small sample of four pastoralist women makes it hard to both draw comparisons between participants and to generalize our findings to other pastoralist women living in these counties, or in Kenya more generally. There could have been errors in how questions were asked, interpreted, and/or recorded, particularly in the interpretation of decision makers and influencers. Recalling important people may also have been challenging for some participants [85]. In addition, missing data will have affected how we interpreted findings from an already small dataset. However, missing data was relatively uncommon and to deal with data quality concerns, we ensured that interviews were conducted in pairs, while one interviewed, the other took notes. We also carried out quality checks throughout the day and further validated data by sharing findings and our interpretation of the data with local Ministry of Health and Save the Children staff members, in addition to community members.

Some challenges are specific to conducting research amongst these nomadic and semi-nomadic pastoralist communities. A potential limitation of the work is the small sample size of four egos originally interviewed. We also restricted the number of alters they could name to five, which has limitations [86, 87]. Future work could interview a wider range of egos, and potentially set no limits on the alters they can name, this would potentially allow for further generalisations to be made. The extremely sensitive nature of discussing FP in these communities limited the questions we could ask. We were unable to ask about current use, which would have been important for exploring how support amongst the reference group affected use. The taboo nature of modern FP may also have influenced participant answers. Comparisons across reference groups, according to age groups may have been artificial, as differences likely exist by life stages and the number of children an individual has. Finally, social networks may have been influenced by the living arrangements of participants at the time of data collection, and as such our study does not reflect the potentially changing nature of pastoralist women’s social networks. Future studies could explore whether and how these networks change in response to migration and herding patterns, during which younger and older men are away for varying periods of time [88] or when access to information and services is better due to temporarily settling closer to towns. Despite these limitations, the in-depth studies of these four pastoralist women allowed us to unpack their complex social networks and how these relate to modern FP. This study provides a useful guide for future research to explore how perceived support impacts uptake of modern FP, how social networks of pastoralists change with migration, and to further explore using social network methods amongst these populations, or similar groups.

### Implications for practice

This research has several implications for practice. We show the importance of using network methods to understand RSH decision making, particularly from the perspective of individual women. Importantly, using an egocentric approach these networks emerged from the women themselves, rather than the researchers imposing predefined reference groups. Interventions, including education and awareness raising, can target individuals who are influential in RSH decision making and include life-stage appropriate components. Our findings provide further evidence for including men in RSH interventions, particularly as husbands are key in the decision-making process for women. While the evidence supporting male involvement in reproductive health is well established, this study highlights the value of studying the men’s networks for more targeted interventions. It also revealed some men are supportive of modern FP, including community leaders, who may not traditionally be associated with positive attitudes towards FP. We point towards transitions in RSH influencers over the life course, with mothers influential before a woman marries and female peers and relatives more influential afterwards. Finally, this study indicates an opportunity to use the support for breastfeeding to provide education (through small group engagement) and counselling on exclusive breastfeeding, or LAM, as an entry point to discuss other modern methods of FP.

## Conclusion

Egocentric network approaches prove valuable to exploring reproductive health reference groups, particularly where there exists little prior research. It is likely that pastoralist women’s networks change as a result of periods of migration and conflict. However, we found husbands remained key players in reproductive health decisions, including use of modern FP, despite being away for periods of time. Husbands were always unsupportive of modern FP use. Mothers and female neighbors provide key support and influence in broader RSH issues, with mothers particularly important. Interventions that seek to increase awareness of available modern FP should consider engaging women’s wider network, to engage in discussions around the acceptability of modern FP.

## Data Availability

The datasets generated and analysed for this study are not currently publicly available of the sensitive nature of the qualitative data that can be linked back to individuals. They are available from the author on reasonable request.

## Abbreviations

DHS: Demographic Health Surveys
FP: Family planning
LAM: Lactational amenorrhea method
RSH: Reproductive and sexual health
SDM: Standard days method
SNA: Social network analysis

## Footnotes

1 Where husbands have more children than their wives, this is explained by polygamous relationships.
